# A taxonomic study of Chinese species of the *insidiosus* group of *Metaphycus* (Hymenoptera, Encyrtidae)

**DOI:** 10.3897/zookeys.378.6156

**Published:** 2014-02-07

**Authors:** Ying Wang, Cheng-De Li, Yan-Zhou Zhang

**Affiliations:** 1 Key Laboratory of Zoological Systematics and Evolution, Institute of Zoology, Chinese Academy of Sciences, Beijing 100101, China; 2 School of Forestry, Northeast Forestry University, Harbin, 150040, China

**Keywords:** Chalcidoidea, parasitoids, natural enemy, new species, China

## Abstract

In this paper, twelve *insidiosus*-group species of the genus *Metaphycus* Mercet from China are reviewed. Five species, *M. corniae* **sp. n.**, *M. cylindricus* **sp. n.**, *M. deltoideus* **sp. n.**, *M. transversus* **sp. n.** and *M. yaanensis* **sp. n.**, are described as new to science. A key to the females of these species is given to facilitate species recognition. Photomicrographs are provided to illustrate morphological characters of these species. All specimens, unless otherwise specified, are deposited in the National Zoological Museum of China, Institute of Zoology, Chinese Academy of Sciences, Beijing.

## Introduction

The present work is the second part of a taxonomic study on the genus *Metaphycus* Mercet from China. In an earlier paper (Wang et al. 2013), we studied 11 *Metaphycus* species of the *alberti*-group, all of which have two segments in the maxillary and labial palpi. The *insidiosus*-group includes the species with both maxillary and labial palpi three-segmented (Compere and Annecke 1960). Species of the *insidiosus*-group are important biocontrol agents against soft scales, especially *Coccus* and *Saissetia* (Annecke and Mynhardt 1972, Noyes and Hayat 1994, Guerrieri and Noyes 2000). In the 1950s, *Metaphycus angustifrons* was introduced from China (Taiwan) to the USA (California) for the biological control of brown soft scale, *Coccus hesperidum* Linnaeus (Compere 1957, Dean and Bailey 1960, Kapranas et al. 2007). Regional revisional works concerning this economically important group include Annecke and Mynhardt (1972) (species of South Africa), Viggiani and Guerrieri (1988) (species of Italy), Guerrieri and Noyes (2000) (species of Europe), and Noyes (2004) (species of Costa Rica). As far as we know, only four species of the *insidiosus-group* are recorded from China. The current study aims to enrich the knowledge of Chinese species of the *insidiosus*-group in *Metaphycus* by providing the descriptions, distribution and host records of the species. A dichotomous key to species of this group known from China is also presented.

## Material and methods

Morphological terminology and abbreviations follow those of Noyes (2004) and Wang et al. (2013). Absolute measurements were used for body length. Relative measurements were used for other dimensions and measured with a Motic SMZ-168 stereomicroscope, under 50× magnification, and the absolute measurement of each unit is 0.02 mm. The following abbreviations are used in the text:

BMNH Natural History Museum, London, UK

IZCAS Institute of Zoology, Chinese Academy of Sciences, Beijing, PR China

IEEM Instituto Español de Entomología, Madrid, Spain

USNM United States National Museum, Washington, DC, USA

ZISP Zoological Institute, St. Petersburg, Russia

## Results

### Key to *Metaphycus* species of the *insidiosus*-group (females) from China

**Table:** 

1	Mid and hind tibiae immaculate (Figs 5, 12, 27, 33), completely yellow	2
–	Mid and hind tibiae each with two brown bands, or at least mid tibia subbasally marked with a brownish band (Figs 61, 68, 75, 82)	6
2	Scape flattened and expanded (Figs 1, 8), less than 3× as long as broad	3
–	Scape not flattened and expanded (Figs 15, 22, 29), at least 4× as long as broad	4
3	Scape about 2× as long as broad (Fig. 1); head about 4× as wide as frontovertex; F5 entirely yellowish white	*Metaphycus orientalis* (Compere)
–	Scape about 2.5× as long as broad (Fig. 8); head about 5× as wide as frontovertex; F5 externally marked with dark brown	*Metaphycus angustifrons* Compere
4	Head entirely black	*Metaphycus nitens* (Kurdjumov)
–	Head generally yellow, at most genae and occiput marked dark brown	5
5	Ovipositor about 2× as long as mid tibia (Fig. 28)	*Metaphycus gerardi* Sugonjaev
–	Ovipositor less than 1.5× as long as mid tibia (Fig. 35)	*Metaphycus garmon* Guerrieri & Noyes
6	Ovipositor (Figs 42, 49, 52) a little longer than mid tibia	7
–	Ovipositor (Figs 63, 65, 77, 79) slightly shorter than mid tibia	9
7	Ovipositor (Figs 49, 52) less than 5× as long as gonostylus; F1–F4 subequal in length, each segment about 0.7× as long as wide (Figs 43, 50)	8
–	Ovipositor (Fig. 42) about 6× as long as gonostylus, F1–F4 strongly transverse in length, each segment about 0.4× as long as wide (Fig. 36)	*Metaphycus transversus* sp. n.
8	Scape slightly expanded, 3–4× as long as broad (Fig. 43)	*Metaphycus eriococci* (Timberlake)
–	Scape not expanded, more than 5× as long as broad (Fig. 50)	*Metaphycus cylindricus* sp. n.
9	Scape more than 3.2× as long as broad (Fig. 57)	*Metaphycus yaanensis* sp. n.
–	Scape less than 3× as long as broad (Figs 64, 70, 78)	10
10	Scape (Fig. 64) about 2.7× as long as broad, maximum width located at subapical part of scape; fore wing (Fig. 66) generally with a distinct infuscation below marginal vein; ovipositor clearly exserted	*Metaphycus deltoideus* sp. n.
–	Scape (Figs 70, 78) less than 2.5× as long as broad, maximum width located at the median part of scape; fore wing (Figs 73, 80) hyaline or at most indistinctly infuscate below marginal vein; ovipositor unclearly exserted or hardly exserted	11
11	Ocelli forming an acute angle about 60°; scape (Fig. 70) expanded, about 2× as long as broad; hind tibia (Fig. 76) subbasally marked with a faintly brown ring	*Metaphycus corniae* sp. n.
–	Ocelli forming an acute angle about 30°, clearly less than 60°; antenna with scape (Fig. 78) expanded, about 2.3× as long as broad; hind tibia (Fig. 83) with two distinctly brown rings	*Metaphycus insidiosus* (Mercet)

### Metaphycus orientalis

(Compere)

http://species-id.net/wiki/Metaphycus_orientalis

Figs 1–7

Aphycus orientalis Compere, 1924: 120. Holotype. ♀ (USNM, not examined), Japan.
Metaphycus orientalis ; Flanders and Bartlett 1964: 39–42; Trjapitzin 1975: 9; Trjapitzin 1989: 234.

#### Female.

**Figures 1–7.**
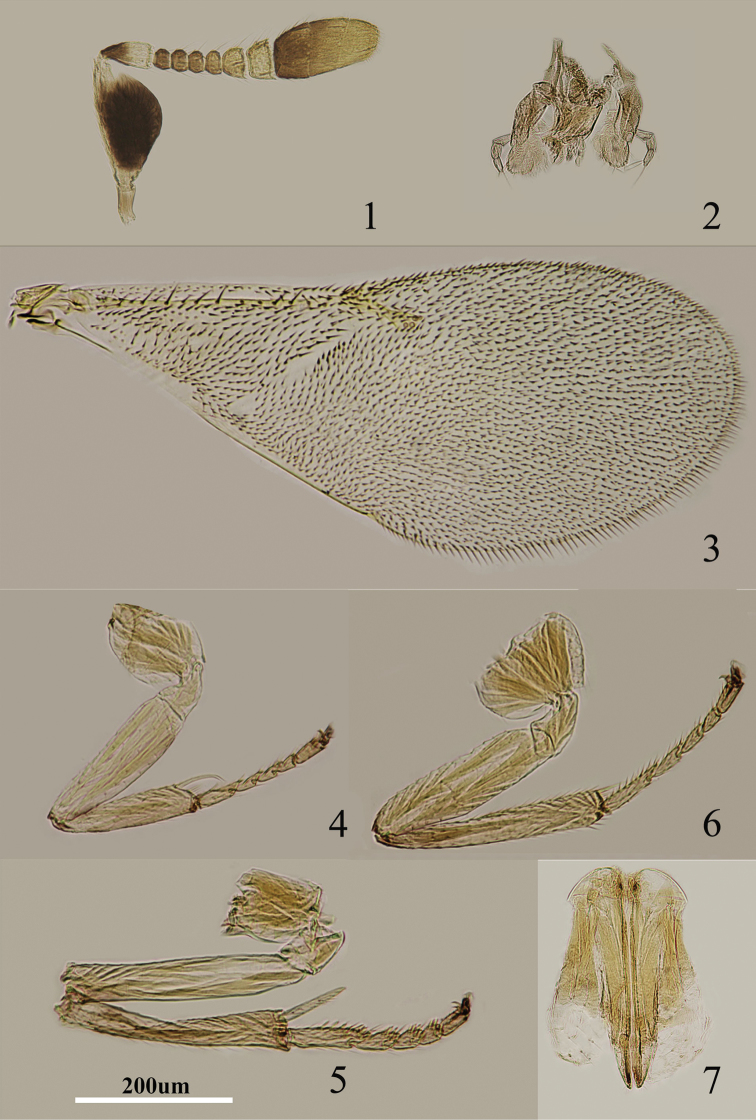
*Metaphycus orientalis* (Compere) Female: **1** antenna **2** palpi **3** fore wing **4** fore leg **5** mid leg **6** hind leg **7** ovipositor.

Body length, including ovipositor, 0.6–0.8 mm. Frontovertex pale orange; immaculate with yellow from occiput to base of mandible; mouth margin medially yellow below torulus; gena yellowish white; antenna (Fig. 1) with radicle pale brown; scape with both faces black, only base and apex white, narrowly white along the dorsal margin; pedicel dark brown in proximal one-third, otherwise white; F1–F4 dark brown, F5–F6 yellow-white; basal segment of clava dark brown, remaining segments becoming slightly paler towards apex, apex yellow-white; occiput with a brown area above foramen, otherwise yellow; neck of pronotum pale brown to dark brown, posterior margin white, lateral spots relatively small and distinct; dorsum of thorax orange; sides and posterior margin of mesoscutum and axillae inconspicuously bordered brown; setae translucent, silvery in most lights; tegula white with apex pale brown; metanotum orange; mesopleuron white; prosternum and mesosternum white; legs (Figs 4–6) pale yellow, tibiae proximally brown, mid tibiae subbasally with an indistinct brown band; fore wing (Fig. 3) hyaline, linea calva interrupted by two to four lines of setae; venation yellow-brown; hind wing hyaline; propodeum medially orange, laterally yellow; gaster dorsally yellow to very slightly pale brown, ventrally white; gonostylus orange.

Head about 4× as wide as frontovertex, head with polygonally reticulate sculpture, mesh size slightly less than size of one eye facet; frontovertex about one-fourth head width; ocelli forming an acute angle of about 30°; eye not quite reaching occipital margin, separated by a little less than diameter of a facet; frontovertex not subparallel, becoming wider anteriorly, narrowest about level with anterior margins of posterior ocelli; scrobes deep, U-shaped; antenna with scape about 1.8× as long as broad; funicle with F1–F4 smallest, F5 a little larger than F4 but transverse, F6 largest and wider than long; linear sensilla only on F5 and F6; clava 3-segmented, its apex more or less rounded but with a short, slightly oblique truncation; mandible relatively broad with three subequal, apical teeth; palpal formula 3-3 (Fig. 2), notaular lines reaching about 0.4× across mesoscutum; fore wing venation and setation as in Fig. 3; cercal plate about in the 1/3 of gaster; ovipositor (Fig. 7) hardly exserted, about 4.8× as long as gonostylus.

Relative measurements: HW 15, FV 3.5, FVL 7, POL 1.5, AOL 3, OOL 0.5, OCL 2.5, POD 1, AOD 1, EL 9, EW 7, MS 5, SL 6, SW 3, FWL 33, FWW 15, HWL 25, HWW 5, OL 13, GL 3, MT 13.

#### Male.

Unknown.

#### Host.

*Coccus hesperidum* Linnaeus, *Coccus pseudomagnoliarum* (Kuwana), *Saissetia coffeae* (Walker) (Noyes 2002).

#### Distribution.

China (Hainan) (Fig. 84); Japan, USA (Biocontrol introduction) (Noyes 2002).

#### Material examined.

China: 2 ♀♀, Hainan, Diaoluo Mt., 6–7.V.2007, Coll. Y. Z. Zhang; 1 ♀, Hainan, Diaoluo Mt., 6.V.2007.

#### Diagnosis.

Scape with both faces black, apices and dorsal margin of scape white; F5 entirely yellowish white (Fig. 1); gena yellowish white; mid tibia subbasally with a faint brown band (Fig. 5); head about 4× as wide as frontovertex; scape about 1.8× as long as broad (Fig. 1); ovipositor (Fig. 7) hardly exserted, about 4.8× as long as gonostylus. *Metaphycus orientalis* is similar to *Metaphycus angustifrons* in general appearance but can be separated from the latter by the scape about 1.8× as long as broad (in *angustifrons*, scape about 2.4× as long as broad); head about 4× as wide as frontovertex (in *angustifrons*, head about 5× as wide as frontovertex); F5 entirely yellowish white (in *angustifrons*, F5 marked with brown externally).

### Metaphycus angustifrons

Compere

http://species-id.net/wiki/Metaphycus_angustifrons

Figs 8–14

Metaphycus angustifrons Compere, 1957: 227–229. Holotype ♀ (USNM) U. S. A. Taiwan. examined part (BMNH).
Metaphycus angustifrons Compere; Tachikawa 1963: 185, 186, 189, 190; Trjapitzin 1975: 9; Trjapitzin 1989: 236.

#### Female.

**Figures 8–14.**
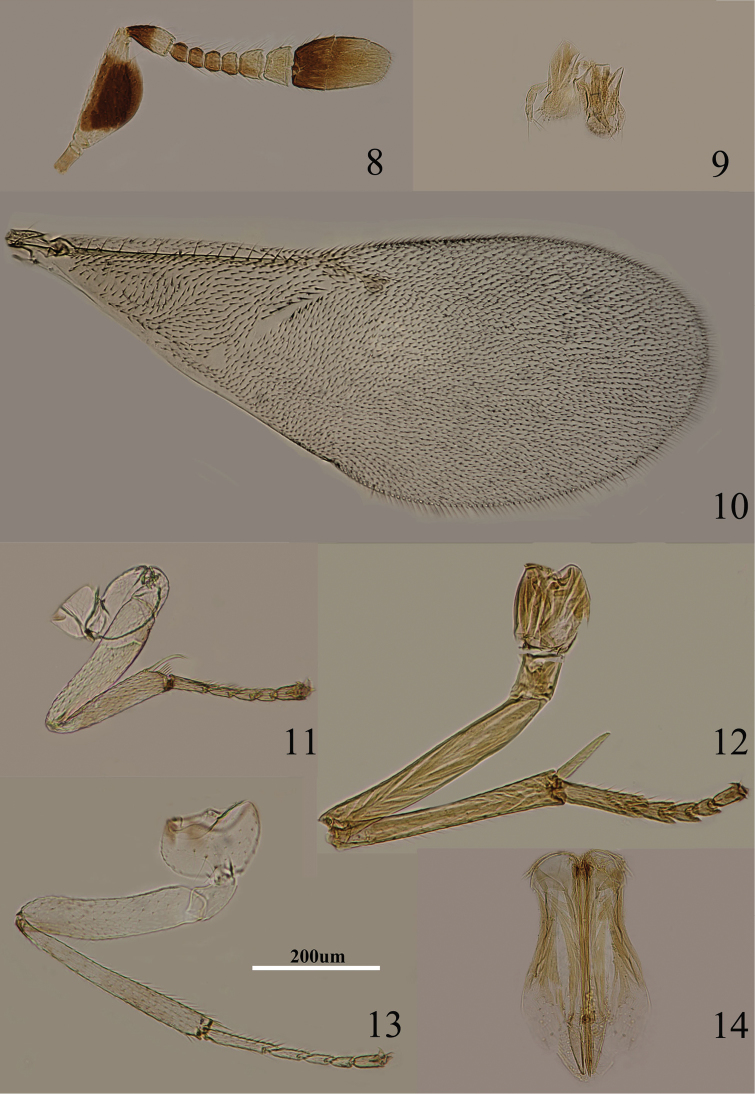
*Metaphycus angustifrons* Compere Female: **8** antenna **9** palpi **10** fore wing **11** fore leg **12** mid leg **13** hind leg **14** ovipositor.

Body length including ovipositor about 1 mm; frontovertex orange, lower face and gena concolorous, paler yellow; antenna (Fig. 8) with radicle pale brown; scape mostly brown with similar coloration on inner and outer surfaces, apex and base white, dorsal margin white; pedicel yellowish white in apical two-fifths, remainder dark brown; F1–F4 brown, F5 marked with brown externally, F6 white; clava dark brown, apical half paler yellow-brown, more or less yellow apically; occiput dark brown dorsally; neck of pronotum blackish, rest of pronotum pale orange, lateral spots present and faint; mesoscutum and scutellum dusky orange, more or less blackish on it, darker than frontovertex; metanotum largely dark brown; legs (Figs 11–13) including coxae pale orange except for faint dots at base of middle tibia; wing hyaline (Fig. 10), venation pale brown; middle of propodeum dark brown, sides orange; gaster dorsally brown.

Head about 5× as wide as frontovertex; head with fine, reticulate sculpture on frontovertex; ocelli forming an acute angle; frontovertex narrowest slightly in front of posterior ocelli; antenna (Fig. 8) with scape about 2.4× as long as broad; F1–F4 relatively small, subequal in size, F5 larger than F4, F6 largest and widest, F5 and F6 with linear sensilla; apex of clava more or less rounded, hardly with transverse truncation; mandible relatively broad with three, apical teeth; palpal formula 3-3 (Fig. 9); fore wing with venation and setation as in Fig. 10; gaster with ovipositor (Fig. 14) slightly exserted, exserted part about 0.3× as long as mid tibial spur; ovipositor about 5× as long as gonostylus.

Relative measurements: HW 16, FV 3, FVL 8, POL 0.5, AOL 2, OOL 0.5, OCL 0.5, POD 1, AOD 1, SL 6, SW 3, FWL 36, FWW 15, OL 14, GL 3, MT 13.

#### Male.

Scape expanded, and about 2.5× as long as wide; funicle blackish or brown; clava similar to the funicle except for dilute yellowish apically (Compere 1957).

#### Host.

*Coccus hesperidum* Linnaeus, *Coccus pseudomagnoliarum* (Kuwana), *Pulvinaria psidii* Maskell, *Saissetia oleae* (Olivier) (Noyes 2002).

#### Distribution.

China (Fujian, Hainan, Hong Kong) (Fig. 84), Bermuda, Japan, USA (Noyes 2002).

#### Material examined.

China: 1 ♀, Fujian, Youxi, 12.V.1965; 1 ♀, Hainan, 11.VIII.2009, Coll. G. Zheng; USA: 1 ♀, Florida, 12.V.1992, Coll. Patrick Gr.; 2 ♀♀, Ins. C.E.S., 29.III.1954, Coll. Bartlett (BMNH).

#### Diagnosis.

Scape with both faces brown, only base and apex white, dorsal margin white; F5 marked with brown externally (Fig. 8); gena pale yellow; mid tibia with faint brown spot subbasally (Fig. 12); head about 5× as wide as frontovertex; scape about 2.4× as long as broad; ovipositor (Fig. 14) slightly exserted, about 5× as long as gonostylus.

### Metaphycus nitens

(Kurdjumov)

http://species-id.net/wiki/Metaphycus_nitens

Figs 15–21

Aphicus [*sic*] *nitens* Kurdjumov, 1912: 334. Syntypes ♀♂, Ukraine, not examined.
Anaphycus nitens (Kurdjumov); Sugonjaev 1960: 372.
Metaphycus nitens (Kurdjumov); Trjapitzin 1975: 12; Guerrieri and Noyes 2000: 159.

#### Female.

**Figures 15–21.**
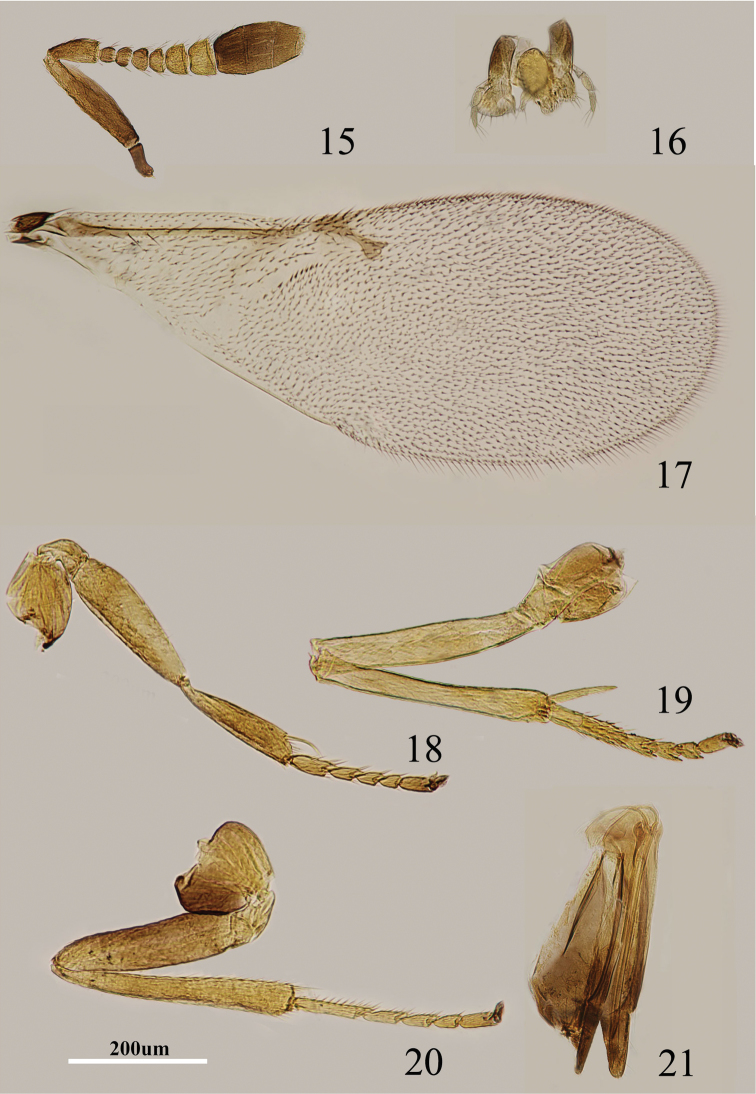
*Metaphycus nitens* (Kurdjumov) Female: **15** antenna **16** palpi **17** fore wing **18** fore leg **19** mid leg **20** hind leg **21** ovipositor.

Body length, including ovipositor, 0.9–1.0 mm. Head black; antenna (Fig. 15) with radicle dark brown; scape brown, apically yellow; pedicel brown in proximal half, otherwise pale brown; F1–F3 brown, F4 very pale brown, F5–F6 brown-yellow, clava dark brown, becoming slightly paler toward apex; dorsum of thorax black; mesoscutum, axillae, scutellum with blue-green reflections, setae translucent pale brown; tegula white with apex black; mesopleuron yellow; prosternum and mesosternum black; legs (Figs 18–20) mainly yellow, but hind femur pale brown; fore wing (Fig. 17) hyaline, linea calva interrupted; venation brown-yellow; hind wing hyaline; propodeum black; gaster black and gonostylus pale yellow-brown.

Head about 3× as wide as frontovertex, head with polygonally reticulate sculpture, mesh size slightly greater than size of one eye facet; frontovertex about one-third head width; ocelli forming an acute angle of about 45°; eye not quite reaching occipital margin, separated by much less than diameter of a facet; frontovertex subparallel; scrobes shallow and U-shaped; antenna with scape 4–5× as long as broad; funicle with F1–F4 smallest, F5 a little larger than F4, F6 largest and wider than long; linear sensilla only on F5 and F6; clava 3-segmented, its apex more or less rounded but with a short, slightly oblique truncation; mandible relatively broad with three subequal, apical teeth; palpal formula 3-3 (Fig. 16), notaular lines indistinct but almost complete; fore wing venation and setation as in Fig. 17; cercal plate about in the 1/2 of gaster; ovipositor (Fig. 21) hardly exserted, about 4× as long as gonostylus.

Relative measurements: HW 17, FV 5, FVL 11, POL 3, AOL 3.5, OOL 1, OCL 2, POD 1, AOD 1, EL 11, EW 8, MS 4, SL 9, SW 2, FWL 48, FWW 18, HWL 31, HWW 7, OL 18, GL 4, MT 17.

#### Male.

Very similar to female except for antenna and genitalia (Guerrieri and Noyes 2000).

#### Host.

*Eriococcus agropyri* (Borchsenius), *Eriococcus greeni* Newstead, *Eriococcus insignis* Newstead and *Eriococcus obscurus* Hoy (Noyes 2002).

#### Distribution.

China (Shanxi) (Fig. 85); Bulgaria, Croatia, Czechia, Slovakia, Finland, Hungary, Moldova, Russia, Former Yugoslavia, Ukraine (Noyes 2002).

#### Material examined.

China: 2 ♀♀, Shanxi, Wutai Mt., 18.VII.2006, 2500m, Coll. Y. Z. Zhang.

#### Diagnosis.

Body entirely black; scape (Fig. 15) brown with apex yellow; legs mainly yellow, but hind femur generally pale brown (Figs 18–20); gonostylus (Fig. 21) pale yellow-brown; antenna with scape 4–5× as long as broad; ovipositor hardly exserted, about 4× as long as gonostylus. According to Guerrieri and Noyes (2000), in *nitens* the legs are largely brown, but with the base and apex of femora, tibiae and tarsi pale brown, whereas in Chinese specimens, the legs are mainly yellow; only the hind femur pale brown (Figs 18–20).

### Metaphycus gerardi

Sugonjaev

http://species-id.net/wiki/Metaphycus_gerardi

Figs 22–28

Metaphycus gerardi Sugonjaev, 1996: 421–422. Holotype ♀ (ZISP), Vietnam.

#### Female.

**Figures 22–28.**
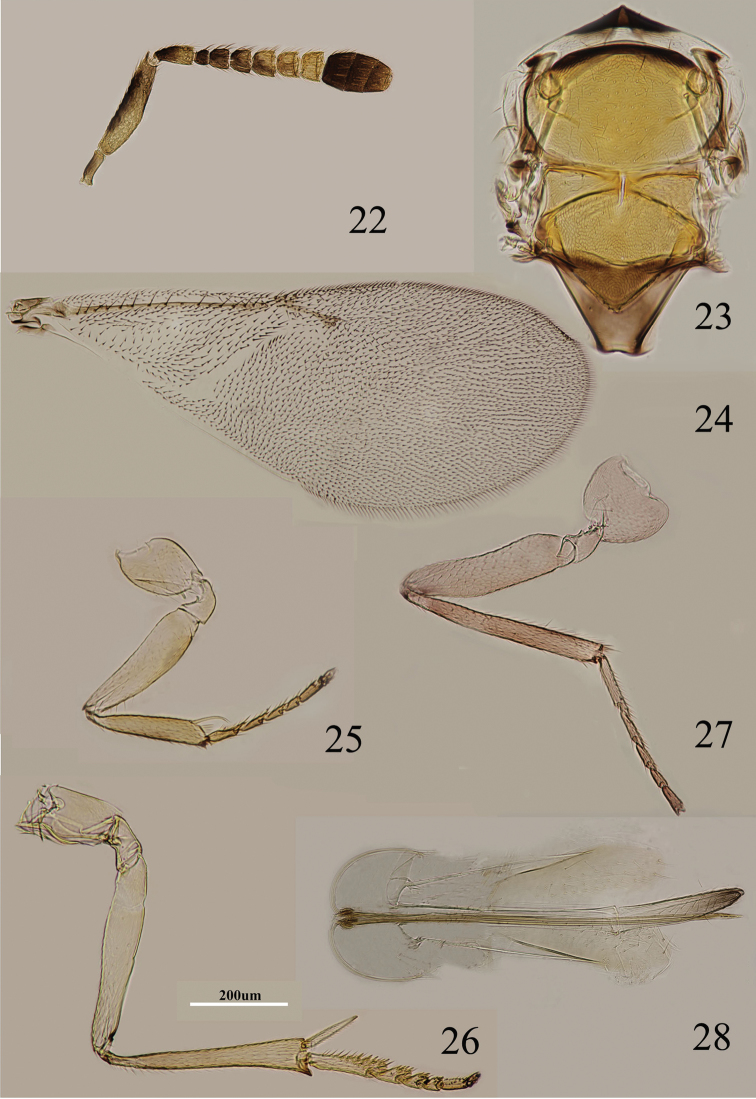
*Metaphycus gerardi* Sugonjaev Female: **22** antenna **23** thorax **24** fore wing **25** fore leg **26** mid leg **27** hind leg **28** ovipositor.

Body length including ovipositor about 1.4 mm. Frontovertex orange; immaculate yellow from occiput to base of mandible; mouth margin below torulus medially yellow; rest of head, except occiput, yellow; antenna (Fig. 22) with radicle yellow; scape not expanded and inner side yellow, outer face yellow but with broad black strip along the dorsal margin; pedicel dark brown in proximal half, otherwise white; F1–F4 dark brown, F5 brown in proximal half, otherwise yellow, brown area extending slightly towards apex, F6 yellow; clava dark brown, apex brown; occiput with brown area above foramen, otherwise yellow; neck of pronotum black, posterior margin brown, otherwise yellow-white, lateral spots relatively large and distinct; dorsum of thorax orange; sides and posterior margin of mesoscutum and axillae inconspicuously pale brown; setae translucent yellow, silvery in most lights; tegula mainly pale brown; metanotum orange; mesopleuron yellow; prosternum, mesosternum yellow-white; legs (Figs 25–27) mainly very pale yellow, only tarsi slightly brown-yellow; fore wing (Fig. 24) hyaline, linea calva almost uninterrupted; venation yellow-brown; hind wing hyaline; propodeum medially orange, laterally black; gaster dorsally orange, side and venter yellow; gonostylus dark brown.

Head about 4× as wide as frontovertex, head with polygonally reticulate sculpture on frontovertex of mesh size about two-thirds size of eye facet; irregular sculpture on frontovertex of rather silky appearance; ocelli forming an acute angle of about 45°; eye reaching occipital margin; frontovertex subparallel and from anterior ocellus slightly wider anteriorly; scrobes deep and U-shaped; antenna with scape 4.0–4.8× as long as broad; funicle with F1 smallest, F2 a little larger than F1, F6 the largest, F2–F6 becoming larger towards apex, F6 slightly wider than long; linear sensilla on F2–F6; clava 3-segmented, its apex rounded; mandible relatively broad with three subequal, apical teeth; palpal formula 3-3, notaular lines reaching about 0.6× across mesoscutum (Fig. 23); fore wing venation and setation as in Fig. 24; cercal plate about in the 0.4× of gaster; ovipositor (Fig. 28) strongly exserted, about 3× as long as gonostylus.

Relative measurements: HW 21, FV 5, FVL 10, POL 3, AOL 4, OOL 0.5, OCL 2, POD 2, AOD 2, EL 13, EW 9, MS 6, SL 8, SW 2, FWL 50, FWW 17, HWL 26, HWW 5, OL 40, GL 13, MT 19.

#### Male.

Length about 1 mm. Dark brown in ocellar area, dark brown between occipital margin and posterior ocelli; scape mainly yellow-white, only dorsal margin of scape with brown; F1 smallest, funicle becoming gradually larger distad with F6 largest; clava solid; dorsum of thorax with black.

#### Host.

*Ceroplastes ceriferus* (Fabricius) (Noyes 2002), *Ceroplastes rubens* Maskell on *Ilex purpurea* Hassk. (new host record), *Ceroplastes rusci* (Linnaeus) on *Ficus microcarpa* Linnaeus (new host record).

#### Distribution.

China (Sichuan, Yunnan) (Fig. 85); Vietnam (Thanh Hoa, Hanoi) (Sugonjaev 1996).

#### Material examined.

China: 2 ♀♀, Sichuan, Panzhihua, 28.IV.2012, Coll. Y. Wang & H.B. Li; 1 ♀, Sichuan, Panzhihua, 2.V.2012, Coll. Y. Wang & H.B. Li; 1 ♀, Yunnan, Kunming, 10.V.2010, Coll. H.L. Shi; 5 ♀♀, 1♂, Yunnan, Kunming, 13.V.2010; 4 ♀♀, 1 ♂, Yunnan, Mengzi (Min’an street), 18.IV.2013. 4 ♀♀, Yunnan, Kunming, 13.V.2013, Coll. J. Deng & X.B. Wang; 1 ♀, Hainan, Diaoluo Mt., 18°09'N, 109°53'E, 930m, 4.V.2007, Coll. Y.Z. Zhang.

#### Diagnosis.

Scape with outer face yellow but with black strip along dorsal margin; scape 4.0–4.8× as long as broad; funicle with F1 smallest, F2–F6 becoming larger towards apex, F2–F6 with linear sensilla (Fig. 22); fore wing hyaline, with linea calva uninterrupted (Fig. 24); ovipositor strongly exserted, and about 2× as long as mid tibia (Fig. 28). *Metaphycus gerardi* is very close to *Metaphycus eruptor* (Howard, 1881) in appearance. They share similar antennal structure, fore wing shape and ovipositor dimensions. Further studies may show they are synonyms.

### Metaphycus garmon

Guerrieri & Noyes

http://species-id.net/wiki/Metaphycus_garmon

Figs 29–35

Metaphycus garmon Guerrieri and Noyes 2000: 181. Holotype ♀ (BMNH), Italy.

#### Female.

**Figures 29–35.**
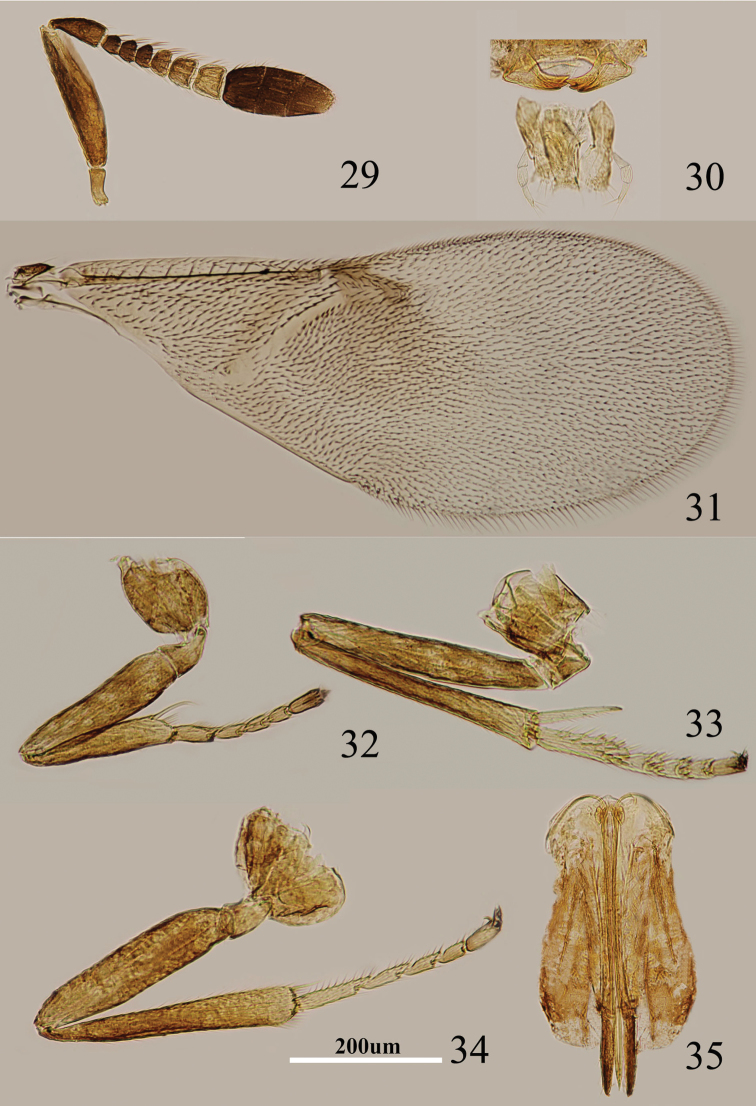
*Metaphycus garmon* Guerrieri & Noyes Female: **29** antenna **30** palpi **31** fore wing **32** fore leg **33** mid leg **34** hind leg **35** ovipositor.

Body length, including ovipositor, 0.8–1.2 mm. Head orange yellow; antenna (Fig. 29) with radicle brown except pale base; scape brown yellow with an elongate black strip on dorsal margin on outer surface except base; pedicel pale yellow in apical half, dark brown in basal half; F1–F3 black, F4 pale brown and becoming progressively paler to apex, F5–F6 white, clava black; neck of pronotum dark brown, posterior margin white, lateral spots relatively small but distinct; mesoscutum and scutellum orange, sometimes anterior margin of mesoscutum brown; sides and posterior margin of mesoscutum and axillae conspicuously bordered brown; setae translucent, silvery in most lights; tegula white with apex brown; metanotum orange; mesopleuron yellow; prosternum and mesosternum pale yellow; legs (Figs 32–34) mainly pale yellow except pretarsus brownish; fore wing infuscate in basal 3/5, with a darker area beneath marginal vein; venation yellow-brown; hind wing hyaline; propodeum medially orange; gaster orange, gonostylus orange.

Head about 4× as wide as frontovertex, head with polygonally reticulate sculpture, mesh size slightly less than size of one eye facet; frontovertex about one-fourth head width; ocelli forming an acute angle about 40°; eye not quite reaching occipital margin, separated by much less than diameter of a facet; frontovertex subparallel and from anterior ocellus slightly wider anteriorly; scrobes shallow and U-shaped; antenna with scape 4–5× as long as broad; funicle with F1–F3 smallest, subequal and subquadrate, F4–F6 becoming larger towards apex; linear sensilla only on F5 and F6; clava 3-segmented, its apex rounded; mandible relatively broad with three subequal, apical teeth; palpal formula 3-3 (Fig. 30), notaular lines reaching about 0.7× across mesoscutum; fore wing venation and setation as in Fig. 31; cercal plate about in the 0.6× of gaster; ovipositor (Fig. 35) slightly exserted, 4–5× as long as gonostylus.

Relative measurements: HW 19, FV 5, FVL 8, POL 3, AOL 4, OOL 0.5, OCL 2, POD 1, AOD 1, EL 11, EW 9, MS 4, SL 10, SW 2, FWL 36, FWW 17, HWL 31, HWW 7, OL 16, GL 4, MT 18.

#### Male.

Generally very similar to female except for body length, coloration of antenna, genitalia and solid clava. Antenna flagellum generally pale brown; aedeagus with two digital spines.

#### Host.

Unknown.

#### Distribution.

China (Beijng, Jiangsu, Shanxi) (Fig. 85); France, Greece, Italy, Spain, Turkey (Guerrieri and Noyes 2000).

#### Material examined.

China: 1 ♀, Beijing, Haidian, 20.VI.2010, Coll. D. K. Zhou; 1 ♀, Beijing, Changping, 20.IX.2008, Coll. F Yuan; 1 ♀, Jiangsu, Nanjing, 18.VI.2012; 1 ♂, Jiangsu, Nanjing, VI. 2011; 2 ♀♀, Shanxi, Wutai Mt., 16.VII.2006, Coll. Y. Z. Zhang

#### Diagnosis.

Scape brown yellow, the outer surface with an elongate black strip along dorsal margin (Fig. 29); fore wing infuscate in basal 3/5, with a darker area beneath marginal vein; scape 4–5× as long as broad; ovipositor slightly exserted, 4–5× as long as gonostylus (Fig. 35). This species is similar to *Metaphycus petitus* in general coloration, antennal structure and ovipositor length. *Metaphycus garmon* can be separated from *Metaphycus petitus* by the coloration of the fore wing and black strip along dorsal margin (Guerrieri and Noyes 2000).

### Metaphycus transversus sp. n.

http://zoobank.org/B5355014-6542-4EE6-B1B2-D555F79F1FE2

http://species-id.net/wiki/Metaphycus_transversus

Figs 36–42

#### Holotype.

China: ♀, Yunnan, Xishuangbanna, 2009.XI.16, Coll. Y. Z. Zhang (IZCAS).

#### Paratypes.

3 ♀♀, the same as holotype (IZCAS).

#### Female.

**Figures 36–42.**
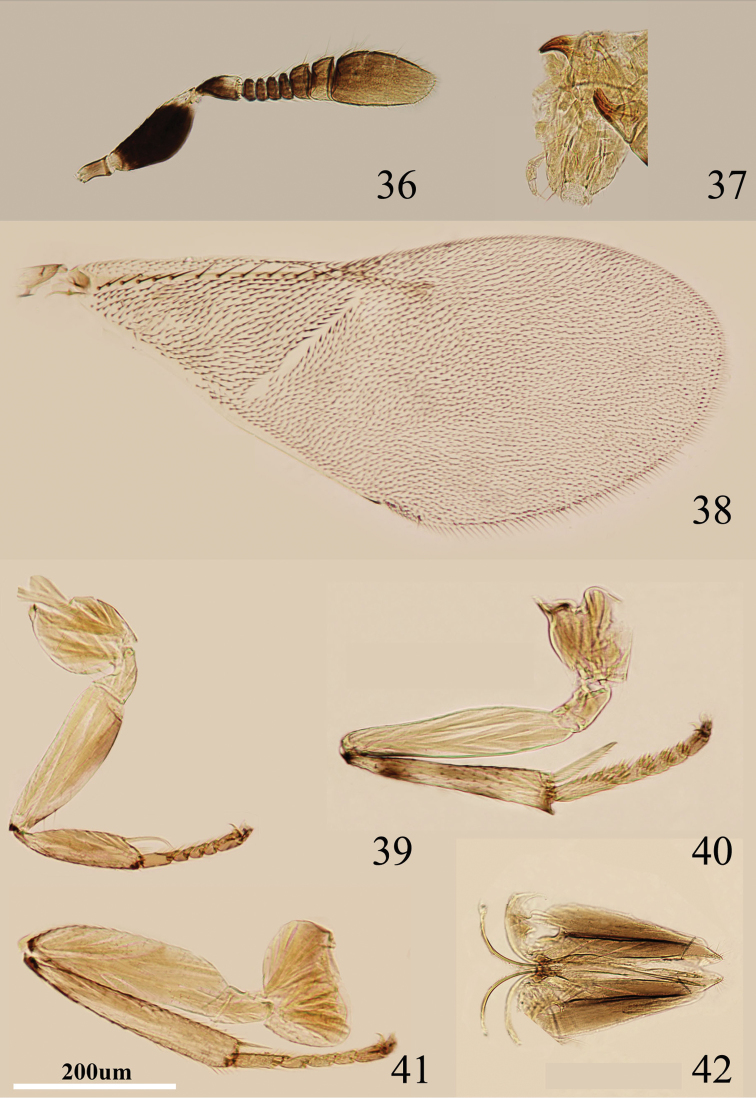
*Metaphycus transversus* sp. n. Female: **36** antenna **37** palpi **38** fore wing **39** fore leg **40** mid leg **41** hind leg **42** ovipositor.

Body length, including ovipositor, 1.2–1.3 mm. Frontovertex pale yellow; gena with fairly broad, oblique brown area near mouth margin; mouth margin medially pale yellow below torulus; rest of head, except occiput, white; antenna (Fig. 36) with radicle brown; scape with both faces black, dorsal margin black, white at extreme apex; pedicel dark brown in proximal half, white distally, dark brown area extending slightly towards apex externally and internally; F1–F4 brown, F5–F6 pale brown and becoming slightly paler towards apex; clava pale brown, extreme base brown like F6, becoming slightly paler towards apex; occiput with a black area above foramen, otherwise white; neck of pronotum black, posterior margin very pale brown, lateral spots relatively small and distinct; dorsum of thorax mainly orange; sides and posterior margin of mesoscutum and axillae conspicuously bordered brown; setae translucent brown, silvery in most lights; tegula pale yellow; metanotum brown; mesopleuron yellow-white; prosternum white; mesosternum white, sometimes pale brown; legs (Figs 39–41) mainly pale yellow; tibiae proximally dark brown; mid tibia and hind tibia with a pair of dark brown rings at about 0.2× and 0.5×; fore wing (Fig. 38) hyaline, with linea calva interrupted by two setae; venation yellow-brown; hind wing hyaline; propodeum medially dark brown, sides pale yellow; gaster dorsally brown, side and venter white; gonostylus orange.

Head about 4× as wide as frontovertex, head with polygonally reticulate sculpture and mesh size slightly less than size of one eye facet; frontovertex about one-fourth head width; ocelli forming an acute angle about 60°; eye not quite reaching occipital margin, separated by much less than diameter of a facet; frontovertex subparallel and from anterior ocellus slightly wider anteriorly; scrobes shallow; antenna (Fig. 36) with scape about 2.5× as long as broad; funicle with all funicular segments transverse, F1–F4 small, F5 and F6 a little larger, linear sensilla only on F5 and F6; clava 3-segmented, its apex rounded but with a short slightly oblique truncation; mandible relatively broad with three subequal, apical teeth; palpal formula 3-3 (Fig. 37), notaular lines reaching about 0.8× across mesoscutum; fore wing venation and setation as in Fig. 38; cercal plate about in the 1/2 of gaster; ovipositor (Fig. 42) hardly exserted, about 5.8× as long as gonostylus.

Relative measurements: HW 19, FV 5, FVL 8, POL 2.5, AOL 2.5, OOL 1.5, OCL 1, POD 1.5, AOD 1.5, EL 11, EW 8, MS 5, SL 7, SW 2.5, FWL 43, FWW 18, HWL 31, HWW 7, OL 15, GL 3, MT 14.

#### Male.

Unknown.

#### Host.

Unknown.

#### Distribution.

China (Yunnan) (Fig. 84).

#### Etymology.

The new species name is derived from the fact that each funicle segment is strongly transverse.

#### Diagnosis.

Scape black, but apex yellowish white; gena with a fairly broad, oblique brown area near mouth margin; funicle with F1–F6 transverse (Fig. 36); ovipositor (Fig. 42) hardly exserted, about 5.8× as long as gonostylus. Using the key of Guerrieri and Noyes (2000), *Metaphycus transversus* runs couplet 27 and is close to *Metaphycus lounsburyi*. Females of *Metaphycus transversus* can be separated from *lounsburyi* as follows: scape (Fig. 36) about 2.5× as long as broad (in *lounsburyi*, scape about 3× as long as broad); dorsal margin of scape black (in *lounsburyi*, dorsal margin white).

### Metaphycus eriococci

(Timberlake)

http://species-id.net/wiki/Metaphycus_eriococci

Figs 43–49

Aphycus eriococci Timberlake, 1916: 631. Holotype ♀, USNM.
Metaphycus eriococci ; Tachikawa 1968: 111.

#### Female.

**Figures 43–49.**
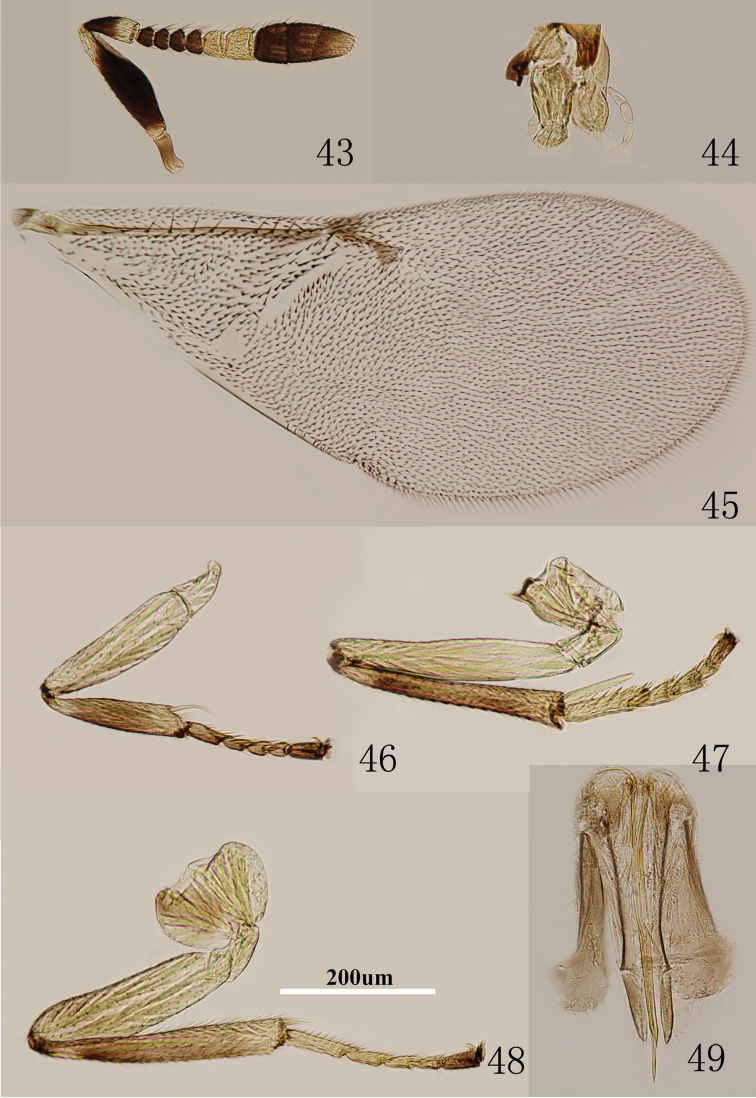
*Metaphycus eriococci* (Timberlake) Female: **43** antenna **44** palpi **45** fore wing **46** fore leg **47** mid leg **48** hind leg **49** ovipositor.

Body length, including ovipositor, 0.7–0.9 mm. Frontovertex pale orange; gena yellow to brownish yellow, gena with brown stripe extending to upper mouth margin; mouth margin medially yellow below torulus; rest of head, except occiput, yellow; antenna (Fig. 43) with radicle dark brown; scape with both faces dark brown, only apex yellowish; pedicel dark brown in proximal half, otherwise yellowish; F1–F4 dark brown, F5–F6 brownish yellow, clava dark brown, becoming slightly paler towards apex and apex very pale brown; occiput with a brown area above foramen, otherwise white; neck of pronotum dark brown to black, posterior margin white, lateral spots relatively large and distinct; dorsum of thorax orange to pale brown; sides and posterior margin of mesoscutum and axillae conspicuously bordered dark brown; setae translucent, silvery in most lights; tegula white with apex pale brown; metanotum orange to brown; mesopleuron yellow-white; prosternum and mesosternum pale yellow; legs (Figs 46–48) mainly pale yellow, mid tibia and hind tibia with faintly brown mark; fore wing hyaline, linea calva interrupted; venation yellow-brown; hind wing hyaline; propodeum medially orange-brown, laterally yellow; dorsum of gaster pale brown and ventral yellow-white; gonostylus orange.

Head about 4× as wide as frontovertex, head with polygonally reticulate sculpture and mesh size slightly less than size of one eye facet; ocelli forming an acute angle about 50°; eye not quite reaching occipital margin, separated by much less than diameter of a facet; frontovertex subparallel and from anterior ocellus slightly wider anteriorly; scrobes shallow and U-shaped; antenna (Fig. 43) with scape 3.2–3.7× as long as broad; funicle with F1–F4 smallest, F5 a little larger than F4, F6 largest; linear sensilla only on F5 and F6; clava 3-segmented, its apex more or less rounded but with a short, slightly oblique truncation; mandible relatively broad with three subequal, apical teeth; palpal formula 3-3 (Fig. 44), notaular lines reaching about 0.6× across mesoscutum; fore wing venation and setation as in Fig. 45; cercal plate about in the 1/2 of gaster; ovipositor (Fig. 49) slightly exserted, 4–5× as long as gonostylus.

Relative measurements: HW 17, FV 4.5, FVL 9, POL 2, AOL 3, OOL 1, OCL 2, POD 1, AOD 1, EL 12, EW 10, MS 5, SL 9, SW 3, FWL 45, FWW 20, OL 17, GL 5, MT 15.

#### Male.

Body length 0.7–0.8 mm, dark brown in coloration. Otherwise very similar to female but for antenna and genitalia.

#### Host.

*Eriococcus howardi* Ehrhorn; *Eriococcus quercus* (Comstock), *Coccus hesperidum* (Linnaeus) (Noyes 2002); and *Eriococcus lagerstroemiae* Kuwana on pomegranate (new host record).

#### Material examined.

China: Beijing, Haidian: 23 ♀♀, 4.IV.2006, Coll. Y. Z. Zhang; 22 ♀♀, 2 ♂♂, 6.VI.2006, Coll. Y. Z. Zhang; 20 ♀♀, 5 ♂♂, 11.VIII.2003, Coll. Y. Z. Zhang; 29 ♀♀, 8 ♂♂, ex *Eriococcus lagerstroemiae* on pomegranate, 12.IX.2006, Coll. Y. Z. Zhang; 19 ♀♀, 1 ♂, ex *Eriococcus lagerstroemiae* on pomegranate, 8.X.2004, Coll. Y. Z. Zhang; 1 ♀, 17.VII.2012, Coll. Q. S. Zhou; 1 ♀, Nanjing, V.2010.

#### Distribution.

China (Beijing, Jiangsu); USA (California, Florida, Texas, Utah) (Noyes 2002) (Fig. 84).

#### Diagnosis.

Antenna with radicle dark brown; scape with both faces dark brown, only apex yellowish; scape 3.2–3.7× as long as broad (Fig. 43); ovipositor (Fig. 49) slightly exserted, 4–5× as long as gonostylus. The Chinese material examined almost agrees with the original description of *eriococci* by Timberlake (1916). The female specimens here have the scape 3.2–3.7× as long as broad, while in original description of *eriococci* the scape is a little over 4× as long as wide (Timberlake 1916).

### Metaphycus cylindricus sp. n.

http://zoobank.org/A3785B09-DD24-42D0-AAAB-ACF69ADF87A9

http://species-id.net/wiki/Metaphycus_cylindricus

Figs 50–56

#### Holotype.

China: ♀, Hunan, Chenzhou, 13.IV.2012. Coll. Y. Wang, J. Deng, H.B. Li (IZCAS).

#### Paratypes.

25 ♀♀, 3 ♂♂, same as holotype (IZCAS).

#### Female.

**Figures 50–56.**
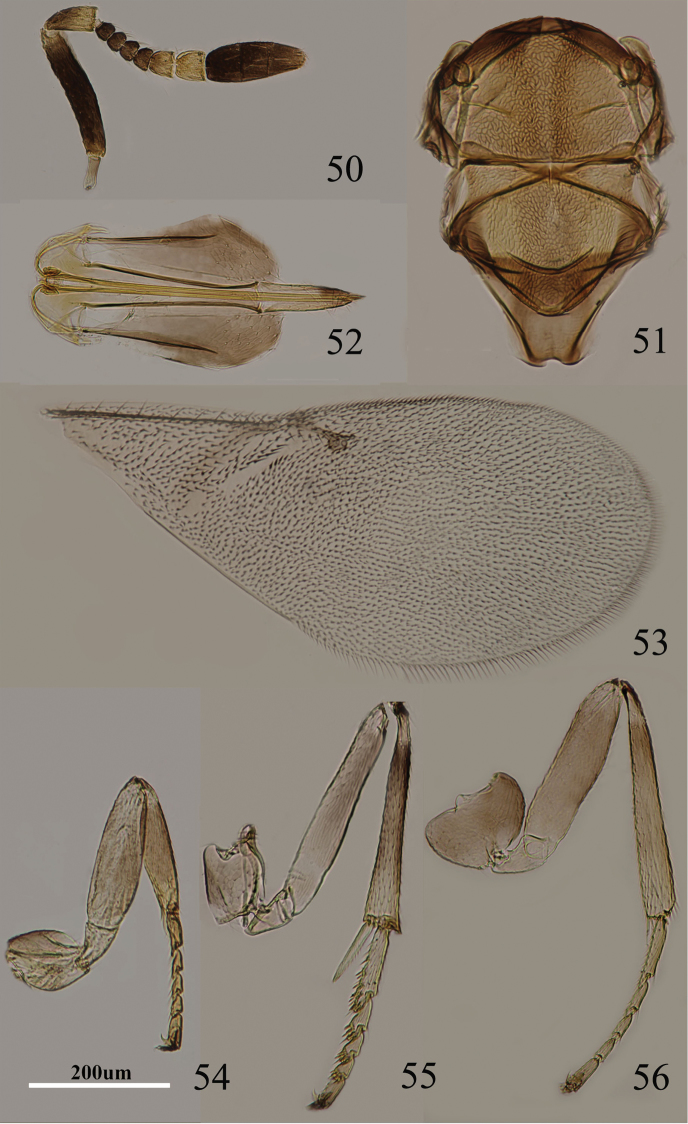
*Metaphycus cylindricus* sp. n. Female: **50** antenna **51** mesoscutum **52** ovpositor **53** fore wing **54** fore leg **55** mid leg **56** hind leg.

Body length, including ovipositor, 1 mm. Frontovertex yellow-brown; occiput largely dark brown; gena and face brown; dark brown on mouth margin below torulus; antenna (Fig. 50) with radicle brown; scape black, only extreme apex slightly yellow; pedicel with proximal half dorsally and laterally brown, remainder yellow; funicle with F1–F4 dark brown, F5–F6 pale yellow; clava dark brown, apical segment yellow; neck of pronotum black, remainder yellow, posterior margin more or less translucent pale yellow, lateral spots present and distinct; mesoscutum, axillae and scutellum brown; dorsum of thorax clothed in scattered, short, translucent setae; tegula proximally pale brown, apex brown; side and venter of mesosoma pale brown; legs brown-yellow (Figs 54–56); mid tibia with faintly brown mark (Fig. 55); fore wing hyaline (Fig. 53), venation pale yellow-brown; propodeum medially brown; gaster mainly brown; gonostylus pale orange.

Head with reticulate sculpture of mesh size the same as eye facet; ocelli forming an acute angle; frontovertex margins subparallel; antennal scrobes fairly deep, U-shaped and meeting dorsally; torulus separated from mouth margin by less than its own length; antenna with scape subcylindricusl, about 5× as long as broad; funicle with F1–F4 subequal, F5 larger, F6 largest and subquadrate; apex of clava more or less rounded; mandible relatively broad with three, acute, subequal apical teeth; palpal formula 3-3; notaular lines present and reaching more than 0.5× across mesoscutum (Fig. 51); fore wing venation and setation as in Fig. 53, gaster with ovipositor clearly exserted; ovipositor (Fig. 52) about 3.3× as long as gonostylus.

Relative measurements: HW 18, FV 5, POL 3, AOL 3, OOL 1, OCL 2, POD 1, AOD 1, EL 13, EW, 8, MS 4, SL 10, SW 2, FWL 45, FWW 19; HWL 31, HWW 8, OL 20, GL 6, MT 15.

#### Male.

Length about 0.7 mm, almost identical to female but for genitalia, solid clava and all funicle segments pale brown.

#### Host.

*Eriococcus lagerstroemiae* Kuwana.

#### Material examined.

China: 35 ♀♀, 1 ♂, Beijing, Mentougou, 14.VIII.2012, Coll. X. Zhang, Q. S. Zhou; 3 ♀♀, 1 ♂, Shandong, Laizhou, 14.X.2012, Coll. F. Yu.

#### Distribution.

China (Beijing, Hunan, Shandong) (Fig. 85).

#### Etymology.

The new species name is derived from the cylindricusl shape of the scape.

#### Diagnosis.

Frontovertex yellow-brown; gena brown; scape black, extreme apex slightly yellow (Fig. 50); scape cylindricusl, about 5× as long as broad; ovipositor (Fig. 52) about 3.3× as long as gonostylus. *Metaphycus cylindricus* is similar to *Metaphycus piceus* in general coloration, antennal structure and ovipositor length. *Metaphycus cylindricus* can be separated from the latter as follows: ovipositor about 1.4× as long as mid tibia, about 3.3× as long as gonostylus (Fig. 52) (in *piceus*, ovipositor about 1.2× as long as mid tibia, 4–5× as long as gonostylus); mid tibia subbasally marked dark brown (in *piceus*, mid tibia yellowish); antenna with pedicel with basal half brown (in *piceus*, pedicel with basal 2/3 brown).

### Metaphycus yaanensis sp. n.

http://zoobank.org/E8507DAA-D044-4A33-84F4-60C596AF35EB

http://species-id.net/wiki/Metaphycus_yaanensis

Figs 57–63

#### Holotype.

♀, China, Sichuan, Ya’an (Tianquan, Erlang Mt.), 2006. V., Coll. W. Li (IZCAS).

#### Paratypes.

6 ♀♀, 3 ♂♂, the same as holotype (IZCAS).

#### Female.

**Figures 57–63.**
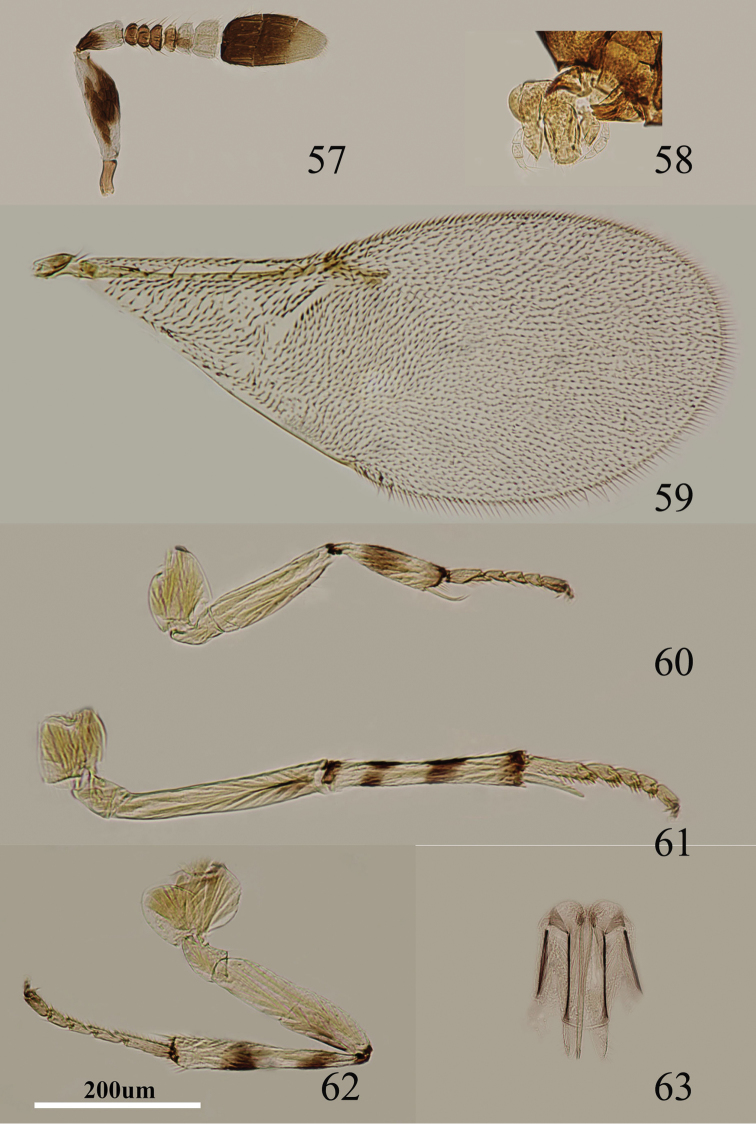
*Metaphycus yaanensis* sp. n. Female: **57** antenna **58** palpi **59** fore wing **60** fore leg **61** mid leg **62** hind leg **63** ovipositor.

Body length, including ovipositor, 1.0–1.2 mm. Yellow in ocellar area, pale orange between occipital margin and posterior ocelli, otherwise pale yellow; lower half of gena with oblique brown stripe, stripe close to scrobe interrupted by a yellow line; upper half yellow; medially yellow below torulus, mouth margin dark brown; rest of head, except occiput, white; antenna (Fig. 57) with radicle dark brown; both faces of scape, dorsal margin, ventral margin with yellow, both sides with broad black strip; pedicel pale yellow (whitish) in apical half, dark brown in basal half; F1–F3 dark brown, F4 very pale brown, F5 venter sometimes with very pale brown strip, F6 white, clava dark brown, becoming slightly paler towards apex, apex very pale brown; occiput brown; neck of pronotum black, posterior margin white, lateral spots relatively large and distinct; dorsum of thorax yellow-brown; sides and posterior margin of mesoscutum and axillae conspicuously bordered dark brown; setae translucent, silvery in most lights; tegula white; metanotum dark brown; mesopleuron very pale yellow, margin of mesopleuron with conspicuously bordered dark brown; prosternum and mesosternum pale brown; legs (Figs 60–62) mainly pale yellow to pale orange; tibiae proximally brown; fore femur yellow, fore tibia with single, broad, interrupted, median dark brown ring; mid tibia and hind tibia with two interrupted conspicuous dark brown rings at 0.2× and 0.7×, extreme apex marked with dark brown; tarsi dusky pale yellow; fore wing (Fig. 59) hyaline, linea calva interrupted; venation pale yellow-brown; hind wing hyaline; propodeum medially brown, blackish, laterally brown-yellow; gaster mainly pale brown, but slightly darker brown dorsally from cercal plates to near apex, gonostylus yellowish, 2^nd^ valvifer and outer plate of ovipositor with brown out margin.

Head about 3× as wide as frontovertex, head with polygonally reticulate sculpture, mesh size slightly less than size of one eye facet; frontovertex about one-third head width; ocelli forming an acute angle of about 30°; eye not quite reaching occipital margin, separated by one or two diameters of a facet; frontovertex parallel; scrobes shallow and U-shaped; antenna with scape 3.2–3.5× as long as broad; funicle with F1–F4 smallest, closely adpressed, F5 a little larger than F4 but transverse, F6 largest; linear sensilla only on F6; clava 3-segmented, its apex more or less rounded but with a short slightly oblique truncation; mandible relatively broad with three subequal, apical teeth; palpal formula 3-3 (Fig. 58), notaular lines reaching about 0.4× across mesoscutum; fore wing venation and setation as in Fig. 59; cercal plate about in the 1/2 of gaster; ovipositor (Fig. 63) slightly exserted, about 5× as long as gonostylus.

Relative measurements: HW 15, FV 5, FVL 9, POL 2, AOL 3, OOL 1, OCL 1.5, POD 1, AOD 1, EL 10, EW 7, MS 4.5, SL 7, SW 2, FWL 40, FWW 18, HWL 25, HWW 5, OL 10, GL 4, MT 12.

#### Male.

Length about 1.2 mm. Virtually identical to female except for genitalia and solid clava. Frontovertex pale yellow but ocellar area brown.

#### Host.

Unknown.

#### Distribution.

China (Sichuan) (Fig. 85).

#### Etymology.

The new species name is derived from the origin of the holotype.

#### Diagnosis.

Scape yellow, both surfaces with an broad black mark in the middle (Fig. 57); lower half of gena with an oblique brown stripe which is interrupted by a yellow line outside of scrobe; mid and hind tibiae with two interrupted dark brown rings (Figs 61–62); scape 3.2–3.5× as long as broad (Fig. 57); ovipositor slightly exserted, about 5× as long as gonostylus (Fig. 63). Using the key of Guerrieri and Noyes (2000), this species runs to couplet 27 and is similar to *Metaphycus lounsburyi* (Howard). It can be separated from *Metaphycus lounsburyi* as follows: scape (Fig. 57) 3.2–3.5× as long as broad (in *lounsburyi*, scape about 2.9× as long as broad); ovipositor about 0.8× as long as mid tibia (in *lounsburyi*, ovipositor as long as mid tibia); clava a little longer than funicle (in *lounsburyi*, clava distinctly shorter than funicle). *Metaphycus yaanensis* sp. n. is also similar to *Metaphycus transversus* sp. n. in appearance, but it can be separated from *transversus* by characters in the key.

### Metaphycus deltoideus sp. n.

http://zoobank.org/8D957C3B-C442-49CF-A149-F0534B676847

http://species-id.net/wiki/Metaphycus_deltoideus

Figs 64–69

#### Holotype.

China: ♀, Beijing Mentougou (Donglingshan), 2008.VIII.28, Coll. F. Yuan (IZCAS).

#### Paratypes.

1 ♀, Beijing (Mentougou): 2011.VI.20; 1 ♀, Beijing Mentougou: 2011. VI. 10–VIII. 6. (IZCAS).

#### Female.

**Figures 64–69.**
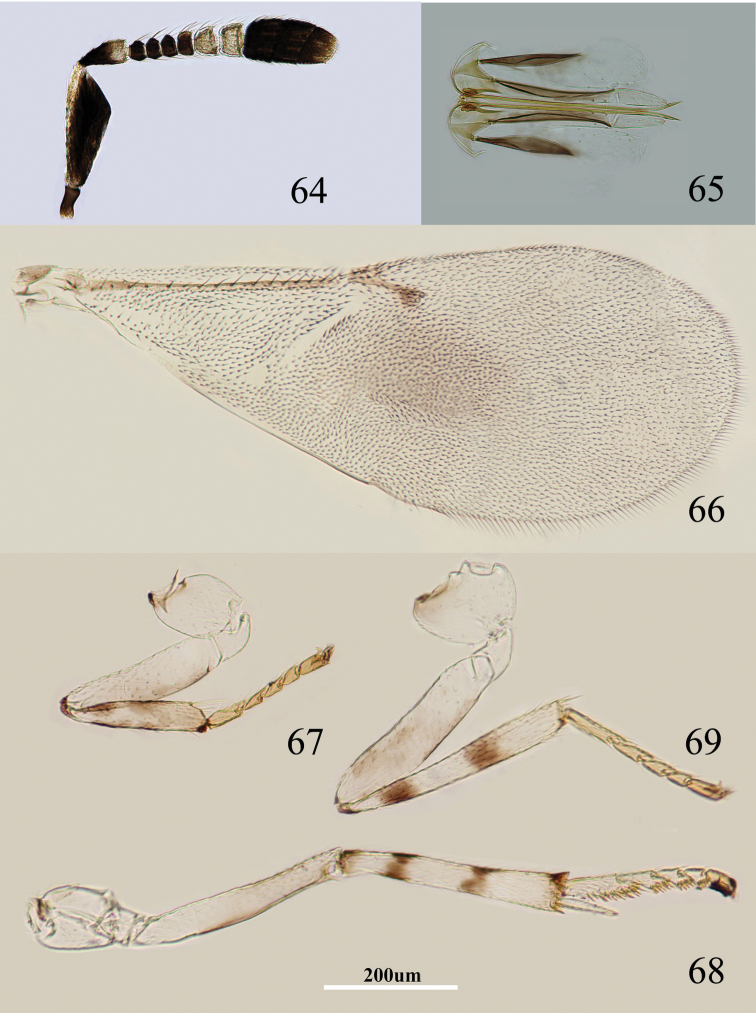
*Metaphycus deltoideus* sp. n. Female: **64** antenna **65** ovipositor **66** fore wing **67** fore leg **68** mid leg **69** hind leg.

Body length, including ovipositor, about 0.9 mm. Frontovertex brownish yellow, but brown between occipital margin and posterior ocelli; gena mainly yellow, with a brown mark extending to oral rim; mouth margin medially yellow below torulus, oral rim brown; antenna (Fig. 64) with radicle dark brown; scape with both faces blackish, and only dorsal margin, venter of base and apex white; pedicel dark brown in proximal half otherwise white; F1–F4 dark brown, F5–F6 white-yellow; clava dark brown, becoming slightly paler towards apex, apex brown; occiput with a brown area above foramen, otherwise yellow; neck of pronotum dark brown, posterior margin translucent yellow, otherwise white, lateral spots relatively large and distinct; dorsum of thorax brown-yellow; sides and posterior margin of mesoscutum and axillae conspicuously bordered dark brown; setae translucent yellow, silvery in most lights; tegula white with apex pale brown; metanotum dark brown; mesopleuron yellowish white; prosternum and mesosternum yellowish white, but with narrow pale brown margin; legs (Figs 67–69) yellowish white, but femur very slightly brown on inner side, tibiae proximally dark brown; each tibia with a pair of dark brown rings at about 0.2× and 0.5× (fore tibia with one faint ring at about 0.5×); fore wing (Fig. 66) hyaline, but generally infuscate below marginal vein; linea calva interrupted by two lines of setae; venation yellow-brown; hind wing hyaline; propodeum medially dark brown, laterally black; gaster dorsally dark brown, side and venter white; gonostylus white.

Head about 4× as wide as frontovertex, head with moderately deep, regular, polygonally reticulate sculpture on frontovertex of mesh size about two-thirds eye facet; irregular sculpture on frontovertex of rather silky appearance; ocelli forming an acute angle of about 30°; eye not quite reaching occipital margin, separated by 1.5× diameter of a facet; frontovertex subparallel; scrobes deep and U-shaped; antenna with scape about 2.7× as long as broad; funicle with F1–F4 smallest, F4–F6 gradually becoming larger distally; linear sensilla only on F5 and F6; clava 3-segmented, its apex more or less rounded but with a short slightly oblique truncation; mandible relatively broad with three subequal, apical teeth; palpal formula 3-3, notaular lines reaching about 0.8× across mesoscutum; fore wing venation and setation as in Fig. 66; cercal plate about in the 1/2 of gaster; ovipositor (Fig. 65) clearly exserted, about 4.5× as long as gonostylus.

Relative measurements: HW 13, FV 3, FVL 10, POL 2, AOL 2.5, OOL 1, OCL 2, POD 1, AOD 1, EL 11, EW 8, MS 3, SL 8, SW 3, FWL 47, FWW 15, OL 14, GL 3, MT 15.

#### Male.

Unknown.

#### Host.

Unknown.

#### Distribution.

China (Beijing) (Fig. 84).

#### Etymology.

The new species name is derived from the shape of the scape.

#### Diagnosis.

Scape with both faces blackish, and only dorsal margin, venter of base and apex white; fore wing hyaline but generally infuscate below marginal vein (Fig. 66); scape triangular in shape, about 2.7× as long as broad (Fig. 64); ovipositor (Fig. 65) about 4.5× as long as gonostylus. Using the key of Guerrieri and Noyes (2000), *Metaphycus deltoideus* runs to couplet 29 and can be separated from *insidiosus* as follow: scape triangular, and strongly expanded subapically, scape with venter of base yellow (Fig. 64) (in *insidiosus*, scape strongly expanded in the median part, scape with venter of base blackish); head mainly yellow-brown (in *insidiosus*, head mainly yellow); fore wing (Fig. 66) with a distinctly infuscate spot below marginal vein (in *insidiosus*, fore wing hyaline or at most slightly infuscate); occiput above foramen brown, rest yellow (in *insidiosus*, occiput almost entirely blackish).

### Metaphycus corniae sp. n.

http://zoobank.org/481A86E7-1FFE-439F-886E-E3658CECCCBC

http://species-id.net/wiki/Metaphycus_corniae

Figs 70–77

#### Holotype.

China: ♀, Beijing, Haidian, 10.V.2013, Coll. S. A. Wu & W. C. Li (IZCAS).

#### Paratypes.

8 ♀♀, 6 ♂♂, same as holotype; 30 ♀♀, Beijing, Chaoyang. 14.V.2013, Coll. X. Zhang (IZCAS).

#### Female.

**Figures 70–77.**
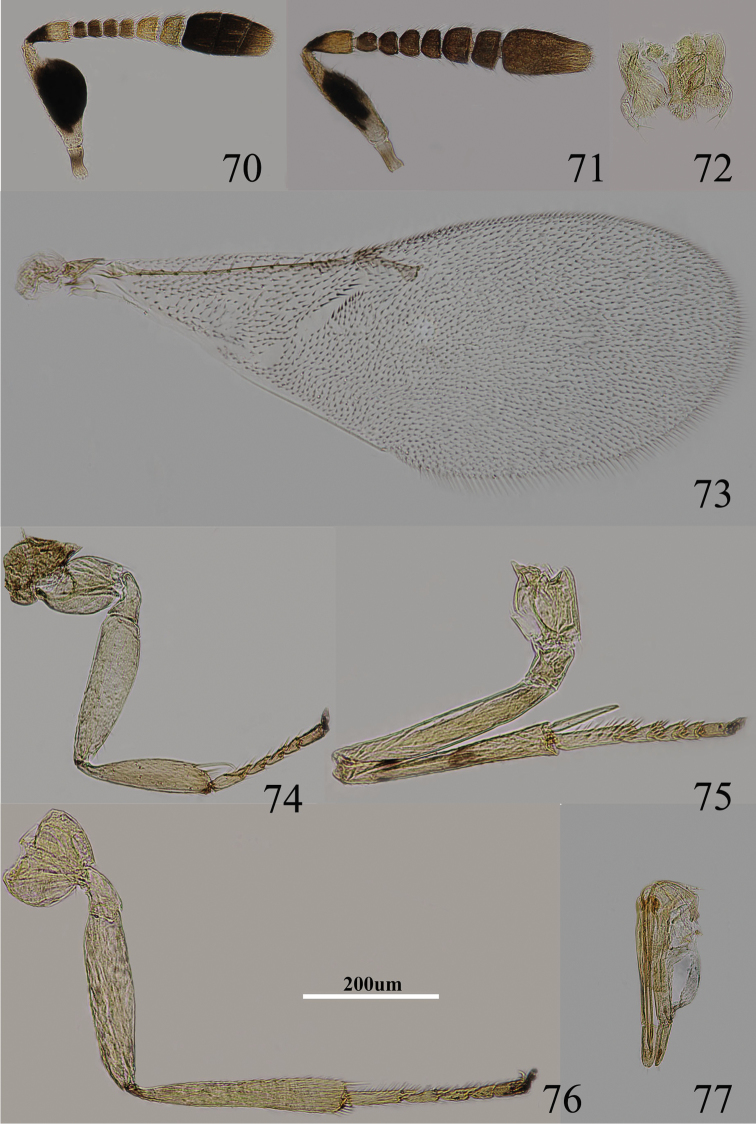
*Metaphycus corniae* sp. n. Female: **70** antenna **72** palpi **73** fore wing **74** fore leg **75** mid leg **76** hind leg **77** ovipositor. male: **71** antenna.

Body length 0.8–1.0 mm. Frontovertex brown, but between occipital margin to posterior ocelli orange, gena yellowish, unmarked; occiput dark brown; antenna (Fig. 70) with scape entirely black, only extreme base and apex white, dorsal margin white; basal half of pedicel black, apex white; F1–F4 blackish, F5–F6 yellowish; clava blackish, but extreme apex brown; pronotum white with a brown spot on each side, mesoscutum, axillae and scutellum pale orange, tegulae white with apex brown, metanotum and propodeum blackish; legs (Figs 74–76) yellowish, proximal tibiae and tarsi brownish, fore tibia and hind tibia with one dark brown ring, mid tibia with two dark brown rings; fore wing hyaline, venation yellow-brown; gaster dorsally black, ventrally white.

Head 3–4× as wide as frontovertex, ocelli forming an acute angle of slightly less than 60°; posterior ocellus closer to occipital margin than eye; antenna with scape expanded, 2.0×–2.3× as long as broad; F1–F4 transverse and F5–F6 gradually increasing in size, F6 largest, linear sensilla present on F5–F6; clava 3-segmented, and apical very slightly oblique; mandible broad with three, subequal apical teeth; palpal formula 3-3 (Fig. 72); notaular lines incomplete and reaching about 0.6× across mesoscutum; fore wing venation and setation as in Fig. 73; cercal plate about in the 1/3 of gaster; ovipositor (Fig. 77) hardly exserted, 4–5× as long as gonostylus.

Relative measurements: HW 16, FV 5, FVL 9, POL 2.5, AOL 3.5, OOL 1, OCL 1.5, POD 1, AOD 1, EL 9, EW 7.5, MS 5.5, SL 7.5, SW 3.5, FWL 47.5, FWW 17.5, HWL 30, HWW 6, OL 13, GL 2.5, MT 15.

#### Male.

Length 0.8–1.0 mm. Generally very similar to female but for relatively narrower scape, coloration of antenna, genitalia and solid clava. Antenna (Fig. 71) with scape 2.8–3.0× as long as broad, flagellum generally pale brown.

#### Host.

*Parthenolecanium corni* (Bouché) on *Fraxinus chinensis* Roxburgh.

#### Distribution.

China (Beijing) (Fig. 84).

#### Etymology.

The new species named for its host “*Parthenolecanium corni* (Bouché)”.

#### Diagnosis.

Scape entirely black, only extreme base and apex white, dorsal margin white (Fig. 70); fore and hind tibiae with one dark brown ring, but mid tibia with two dark brown rings (Figs 74–76); scape expanded, 2.2×–2.3× as long as broad; ovipositor hardly exserted, 4–5× as long as gonostylus (Fig. 77). This species is very similar to *Metaphycus stanleyi*. In *Metaphycus corniae* sp. n. the ovipositor is about 0.9× as long as mid tibia, and 4–5× as long as gonostylus (Fig. 77). In *Metaphycus stanleyi* ovipositor is about 0.7× as long as mid tibia, about 3× as long as gonostylus.

### Metaphycus insidiosus

(Mercet)

http://species-id.net/wiki/Metaphycus_insidiosus

Figs 78–83

Aphycus (Metaphycus) insidiosus Mercet (1921): 218–220. Lectotype ♀ (IEEM, not examined), Spain.
Metaphycus insidiosus ; Mercet (1925): 28; Trjapitzin (1975): 9; Noyes (1981): 168; Viggiani and Guerrieri (1988): 117; Li and Li (2008): 134.
Metaphycus taxi Alam (1957): 426. Synonymy by Noyes (1981): 168.

#### Female.

**Figures 78–83.**
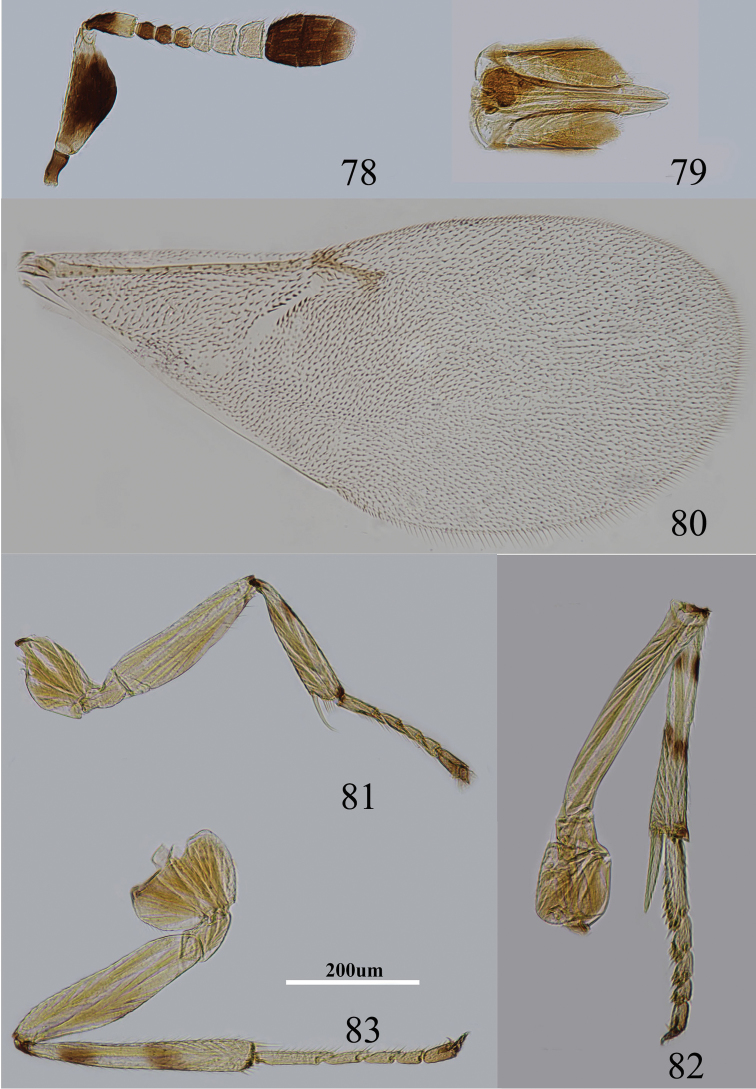
*Metaphycus insidiosus* (Mercet) Female: **78** antenna **79** ovipositor **80** fore wing **81** fore leg **82** mid leg **83** hind leg.

Body length, including ovipositor, about 1.0 mm. Frontovertex yellow; gena with a brown mark extending to oral rim; mouth margin medially pale yellow below torulus; rest of head, except occiput, white; antenna (Fig. 78) with radicle brown; scape with both faces dark brown to blackish, only base, apex white and dorsal margin with a narrow stripe; pedicel dark brown in proximal half, otherwise white; F1–F2 dark brown, F3 slightly pale brown, F4–F6 yellowish; clava dark brown, becoming slightly paler towards apex, extreme apex very pale brown; occiput with a brown area above foramen, rest yellow; neck of pronotum dark, posterior margin white, lateral spots relatively large and distinct; dorsum of thorax pale orange; sides and posterior margin of mesoscutum and axillae conspicuously bordered brown; setae translucent yellow, silvery in most lights; tegula white with apex pale brown; metanotum dark brown; mesopleuron yellow; prosternum yellow and mesosternum pale brown; legs (Figs 81–83) mainly pale yellow, tibiae proximally dark brown; mid tibia and hind tibia with a pair of dark brown rings at about 0.2× and 0.5×, fore tibia with one dark brown ring; fore wing (Fig. 80) hyaline, with linea calva interrupted by several line setae; venation yellow-brown; hind wing hyaline; propodeum medially dark-brown, sides pale yellow; dorsum of gaster largely blackish, sides and ventral parts whitish and gonostylus yellow.

Head about 5× as wide as frontovertex, head with polygonally reticulate sculpture and mesh size as long as one eye facet; ocelli forming an acute angle of about 30°; eye not quite reaching occipital margin, separated by much less than diameter of a facet; frontovertex parallel; scrobes deep and U-shaped; antenna with scape about 2.3× as long as broad; funicle with F1–F3 smallest, F4–F6 gradually increasing in size, F6 largest and slightly wider than long; linear sensilla only on F5 and F6; clava 3-segmented, its apex more or less rounded but with a short slightly oblique truncation; mandible relatively broad with three subequal, apical teeth; palpal formula 3-3, notaular lines reaching about 0.7× across mesoscutum; fore wing venation and setation as in Fig. 80; cercal plate about in the 0.4× of gaster; ovipositor (Fig. 79) slightly exserted, about 4× as long as gonostylus.

Relative measurements: HW 15, FV 3, FVL 9, POL 2, AOL 3, OOL 0.5, OCL 2, POD 1, AOD 1, EL 10, EW 6, MS 5, SL 7, SW 3, FWL 54, FWW 22, OL 12, GL 3, MT 13.

#### Male.

Very similar to female except for antenna, genitalia and generally darker coloration with mesoscutum brownish (Guerrieri and Noyes 2000).

#### Host.

*Eulecanium coryli* (Linnaeus), *Eulecanium taxi* Habib, *Eulecanium tiliae* (Linnaeus), *Parthenolecanium corni* (Bouché), *Parthenolecanium persicae* (Fabricius), *Parthenolecanium pomeranicum* (Kawecki), *Parthenolecanium rufulum* (Cockerell), *Pulvinaria* sp., *Parthenolecanium vitis* (Linnaeus) and *Sphaerolecanium prunastri* (Fonscolombe) (Noyes 2002).

#### Distribution.

China (Heilongjiang) (Fig. 85); Andorra, Armenia, Austria, Azerbaijan, Bulgaria, Canary Islands, Caucasus, Czechia, Denmark, Finland, France, Georgia, mainland Greece, Hungary, Italy, Kazakhstan, Romania, Russia, Slovakia, Spain, Switzerland, United Kingdom (Noyes 2002).

**Figures 84–85.**
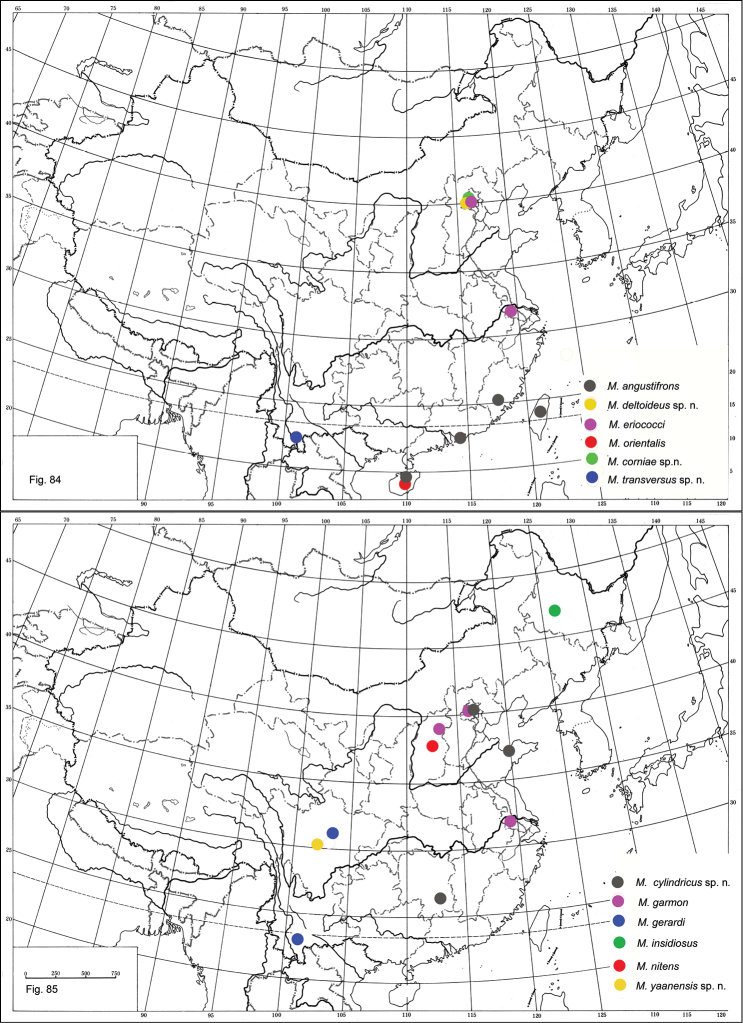
Distribution of *Metaphycus* spp. in China.

#### Material examined.

China: 3 ♀♀, Heilongjiang, Shangzhi, 15.VI.1993, Coll. C.D. Li.

#### Diagnosis.

Scape with both faces dark brown to blackish, only base and apex white and dorsal margin with a narrow stripe; gena with a brown mark extending to oral rim; scape about 2.3× as long as broad (Fig. 78); mid and hind tibiae with a pair of dark brown rings at about 0.2× and 0.5×, fore tibia with one dark brown ring (Figs 81–83); ovipositor slightly exserted, about 4× as long as gonostylus (Fig. 79). According to Guerrieri and Noyes (2000), in *insidious* the fore wing is slightly infuscate, the head is about 4× as wide as frontovertex, and the occiput is almost entirely blackish, whereas in Chinese specimens, the fore wing is hyaline, the head is about 5× as wide as frontovertex, and the occiput has a brown area above the foramen.

## Supplementary Material

XML Treatment for Metaphycus orientalis

XML Treatment for Metaphycus angustifrons

XML Treatment for Metaphycus nitens

XML Treatment for Metaphycus gerardi

XML Treatment for Metaphycus garmon

XML Treatment for Metaphycus transversus

XML Treatment for Metaphycus eriococci

XML Treatment for Metaphycus cylindricus

XML Treatment for Metaphycus yaanensis

XML Treatment for Metaphycus deltoideus

XML Treatment for Metaphycus corniae

XML Treatment for Metaphycus insidiosus

## References

[B1] Alam SM (1957) Taxonomy of some encyrtid parasites (Hymenoptera, Chalcidoidea) of British scale insects. Transactions of the Royal Entomological Society of London 109: 421-466. doi: 10.1111/j.1365-2311.1957.tb00333.x

[B2] Annecke, DP Mynhardt MJ (1972) The species of the *insidiosus*-group of *Metaphycus* Mercet in South Africa with notes on some extra-limital species (Hymenoptera Encyrtidae). Revue de Zoologie et de Botanique Africaines 85: 227-274.

[B3] Compere H (1924) A preliminary report on the parasitic enemies of the citricola scale (*Coccus pseudomagnoliarum* (Kuwana)) with descriptions of two new chalcidoid parasites. Bulletin of the Southern California Academy of Science 23(4): 113-123.

[B4] Compere H (1957) Descriptions of species of *Metaphycus* recently introduced into California and some corrections. Bollettino del Laboratorio di Entomologia Agraria ‘Filippo Silvestri’, Portici 15: 221-230.

[B5] Compere H Annecke DP (1960) A reappraisal of *Aphycus* Mayr, *Metaphycus* Mercet and related genera (Encyrtidae). Journal of the Entomological Society of Southern Africa 23: 375-389.

[B6] Dean HA Bailey JC (1960) Introduction of beneficial insects for the control of *Citrus* scale insects and mites. Journal of the Rio Grande Valley Horticultural Society 14: 40-46.

[B7] Flanders SE Bartlett BR (1964) Observations on two species of *Metaphycus* (Encyrtidae, Hymenoptera) parasitic on citricola scale. Mushi 38(8): 39-42.

[B8] Guerrieri E Noyes JS (2000) Revision of European species of genus *Metaphycus* Mercet (Hymenoptera: Chalcidoidea: Encyrtidae), parasitoids of scale insects. Systematic Entomology 25: 147-222. doi: 10.1046/j.1365-3113.2000.00099.x

[B9] Howard LO (1881) Report of the parasites of Coccidae in the collections of the U.S. Department of Agriculture Part III. Report. United States Department of Agriculture Washington (Entomology) 1880: 350-372.

[B10] Kapranas A Morse JG Pacheco P Forster LD Luck RF (2007) Survey of brown soft scale *Coccus hesperidum* L. parasitoids in southern California *citrus*. Biological Control 42: 288-299. doi: 10.1016/j.biocontrol.2007.05.012

[B11] Kurdjumov NV (1912) Six new species of chalcid flies parasitic upon *Ericoccus greeni* Newstead. Russkoe Entomologicheskoe Obozrenie 12(2): 329-335.

[B12] Li CD Li JW (2008) Description of a new species and two new record species of *Metaphycus* Mercet (Hymenoptera: Encyrtidae) from China. Entomotaxonomia 30(2): 131-139.

[B13] Mercet RG (1921) Fauna Iberica. Himenópteros Fam. Encírtidos. Museo Nacional de Ciencias Naturales, Madrid, 727 pp.

[B14] Mercet RG (1925) El género *Aphycus* y sus afines. Eos, Revista Española de Entomología 1: 7-31.

[B15] Noyes JS (1981) On the types of the species of Encyrtidae described by R. Garcia Mercet (Hymenoptera: Chalcidoidea). Eos, Revista Española de Entomología 55/56: 165–189.

[B16] Noyes JS (2002) Interactive catalogue of World Chalcidoidea, second edition. CDrom, Taxapad, Vancouver and The Natural History Museum, London.

[B17] Noyes JS (2004) *Metaphycus* and related genera, parasitoids of scale insects (Coccoidea) and whiteflies (Aleyrodidae). Encyrtidae of Costa Rica (Hymenoptera: Chalcidoidea). Memoirs of the American Entomological Institute 73(2): 1-460.

[B18] Noyes JS Hayat M (1994) Oriental mealybug parasitoids of the Anagyrini (Hymenoptera: Encyrtidae). CAB International, Oxon, UK, 554 pp.

[B19] Sugonjaev ES (1960) On the species of the genera allied to *Aphycus* Mayr (Hymenoptera, Chalcidoidea) from the European part of the USSR. Entomologicheskoe Obozrenie 39(2): 364-383.

[B20] Sugonjaev ES (1996) Chalcid wasps (Hymenoptera, Chalcidoidea) parasites of soft scales (Coccinea, Coccidae) in Vietnam. IV. New species of the genus *Microterys* Thomson and *Metaphycus* Mercet (Encyrtidae), partly inhabiting ants’ nests, with morphological notes. Entomologicheskoe Obozrenie 75(2): 417-425.

[B21] Tachikawa T (1963) Revisional studies of the Encyrtidae of Japan (Hymenoptera, Chalcidoidea). Memoirs of Ehime University 6(9): 1-264.

[B22] Tachikawa T (1968) A new name for *Metaphycus eriococci* Alam (Hymenoptera, Chalcidoidea - Encyrtidae). Transactions of the Shikoku Entomological Society 9(4): 111.

[B23] Timberlake PH (1916) Revision of the parasitic hymenopterous insects of the genus *Aphycus* Mayr, with notice of some related genera. Proceedings of the United States National Museum 50: 561-640. doi: 10.5479/si.00963801.50-2136.561

[B24] Trjapitzin VA (1975) Contribution to the knowledge of parasitic Hymenoptera of the genus *Metaphycus* Mercet, 1917 (Hymenoptera, Chalcidoidea, Encyrtidae) of the Czechoslovakian fauna. Studia Entomologica Forestalia 2(1): 5-17.

[B25] Trjapitzin VA (1989) Parasitic Hymenoptera of the fam. Encyrtidae of Palaearctics. Opredeliteli po Faune SSSR. Zoologicheskii Institut Akademii Nauk SSR, Leningrad 158: 1-489.

[B26] Viggiani G Guerrieri E (1988) Italian species of the genus *Metaphycus* Mercet (Hymenoptera: Encyrtidae). Bollettino del Laboratorio di Entomologia Agraria ‚Filippo Silvestri‘, Portici 45: 113-140.

[B27] Wang Y Li CD Zhang YZ (2013) A taxonomic study of Chinese species of the *alberti* group of *Metaphycus* (Hymenoptera, Encyrtidae). Zookeys 285: 53-88. doi: 10.3897/zookeys.285.4142 PMC3690757 23798896

